# Peritoneal hydatidosis in an adolescent

**DOI:** 10.11604/pamj.2014.18.210.4905

**Published:** 2014-07-08

**Authors:** Aziz El Madi, Youssef Bouabdallah

**Affiliations:** 1 Department of Pediatric Surgery, Hassan II University Hospital, Fez, Morocco

**Keywords:** Peritoneal hydatidosis, abdominal CT, calcified cyst

## Image in medicine

Hydatid cyst is a common disease in our country. The revelation by peritoneal hydatidosis is rare in children. We report the case of a 15 years old adolescent. History of the disease began a year by the occurrence of acute abdominal pain starting at the right hypochondrium; resolved with symptomatic treatment; no assessment was initially performed. The evolution was characterized by the recurrence of pain increasingly become intense with fever. Clinical examination revealed several abdominal masses spread to the whole abdomen measuring between 3 and 5 cm. Abdominal ultrasound showed peritoneal hydatidosis with splenic, hepatic, Douglas and inter-loop with multiple cysts classified type 1 (classifiations of Gharbi); only one cyst was multivesicular (A); the abdominal CT scan confirmed the same observation, indicated the location of cysts and the presence of a type 5 “calcified cyst” (B). The blood cell counts does not shown any hypereosinophilia and CRP was elevated; a median laparotomy was performed. We proceeded with excision of peritoneal cyst; right hemi-omentectomy because the omentum contained several cysts ranging from 1 to 5 cm in size(C); Sterilizing the content of three hepatic cysts, extracting the germinal membrane; This same technique was realized for both splenic cysts; we have removed all 39 abdominal cavity cysts (D), a drainage was performed by three drains sub-hepatic; splenic lodge and Douglas; the post operative courses was simple; the patient was received albendazole at 10 mg/kg for 6 cycles of 15 days per month. Evolution was favorable.

**Figure 1:**
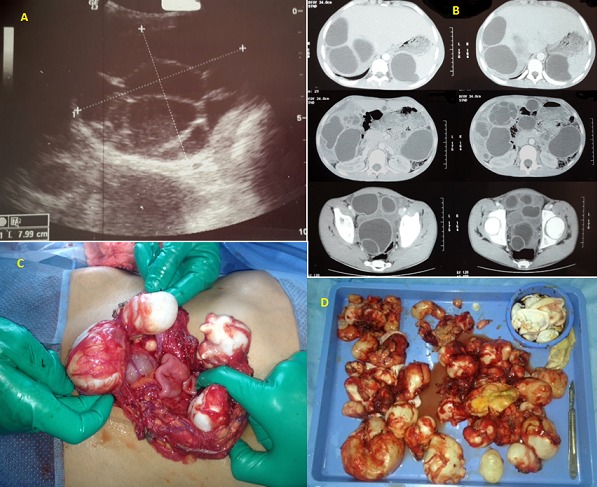
(A) ultrasound image shows a multivesicular hepatic hydatid cyst; (B) CT scan image showing several cysts of the peritoneal cavity; (C) Intraoperative view objectified hydatid cysts of the omentum; (D) all cysts after surgery

